# Neuron-Specific Enolase Is Correlated to Compromised Cerebral Metabolism in Patients Suffering from Acute Bacterial Meningitis; An Observational Cohort Study

**DOI:** 10.1371/journal.pone.0152268

**Published:** 2016-03-28

**Authors:** Jiri Bartek, Eric Peter Thelin, Per Hamid Ghatan, Martin Glimaker, Bo-Michael Bellander

**Affiliations:** 1 Department of Clinical Neuroscience, Section for Neurosurgery, Karolinska University Hospital and Karolinska Institutet, Stockholm, Sweden; 2 Department of Clinical Neuroscience, Karolinska Institutet, Stockholm, Sweden; 3 Department of Infectious Diseases, Karolinska University Hospital, Stockholm, Sweden; 4 Department of Neurosurgery, Copenhagen University Hospital Rigshospitalet, Copenhagen, Denmark; University of Pittsburgh, UNITED STATES

## Abstract

**Introduction:**

Patients suffering from acute bacterial meningitis (ABM) with a decreased level of consciousness have been shown to have an improved clinical outcome if treated with an intracranial pressure (ICP) guided therapy. By using intracranial microdialysis (MD) to monitor cerebral metabolism in combination with serum samples of biomarkers indicating brain tissue injury, S100B and Neuron Specific Enolase (NSE), additional information might be provided. The aim of this study was to evaluate biomarkers in serum and MD parameters in patients with ABM.

**Methods:**

From a prior study on patients (n = 52) with a confirmed ABM and impaired consciousness (GCS ≤ 9, or GCS = 10 combined with lumbar spinal opening pressure > 400 mmH_2_O), a subgroup of patients (n = 21) monitored with intracerebral MD and biomarkers was included in the present study. All patients were treated in the NICU with intracranial pressure (ICP) guided therapy. Serum biomarkers were obtained at admission and every 12 hours. The MD parameters glucose, lactate, pyruvate and glycerol were analyzed. Outcome was assessed at 12–55 months after discharge from hospital. Mann-Whitney U-Test and Wilcoxon matched-pairs signed rank test were applied.

**Results:**

The included patients had a mean GCS of 8 (range, 3–10) on admission and increased ICP (>20 mmHg) was observed in 62% (n = 13/21) of the patients. Patients with a lactate:pyruvate ratio (LPR) >40 (n = 9/21, 43%) had significantly higher peak levels of serum NSE (p = 0.03), with similar, although non-significant observations made in patients with high levels of glycerol (>500 μmol/L, p = 0.11) and those with a metabolic crisis (Glucose <0.8 mmol/L, LPR >25, p = 0.09). No associations between serum S100B and MD parameters were found. Furthermore, median MD glucose levels decreased significantly between day 1 (0–24h) and day 3 (48–72h) after admission to the NICU (p = 0.0001). No correlation between MD parameters or biomarkers and outcome was found.

**Conclusion:**

In this observational cohort study, we were able to show that cerebral metabolism is frequently affected in patients with ABM. Furthermore, patients with high LPR (LPR>40) had significantly higher levels of NSE, suggesting ongoing deterioration in compromised cerebral tissue. However, the potential clinical impact of MD and biomarker monitoring in ABM patients will need to be further elaborated in larger clinical trials.

## Introduction

Acute Bacterial Meningitis (ABM) is a disease associated with high morbidity and mortality [1]. Due to its pathophysiology, ABM may result in increased intracranial pressure (ICP), decreased cerebral blood flow and altered level of consciousness [2, 3]. In order to improve intracranial conditions an ICP targeted therapy (ICP <20 mmHg, cerebral perfusion pressure (CPP) >50 mmHg) through cerebrospinal (CSF) drainage, osmotherapy, hyperventilation, steroids (Methylprednisolone) and sedation has been shown to improve outcome for ABM patients, and has been implemented as a treatment algorithm at our Department [4].

The serum protein biomarkers S100B and NSE are two of the most widely studied in the field of cerebral pathology, functioning as surrogate markers of affected cerebral tissue, thus validating lesion development [5]. S100B is primarily present in mature perivascular astrocytes and is involved in several intra- and extracellular functions [6]. S100B has been extensively studied in traumatic brain injury (TBI) where it is possible to correlate it to cerebral injury and clinical outcome [7]. NSE is present primarily in the cytosol of neurons and is involved in the glycolysis [8]. Serum NSE levels have also been shown to correlate to outcome and injury severity in TBI [9] and has moreover been suggested a better marker of cerebral inflammation than S100B [10]. Nevertheless, up to this date, the number of studies monitoring ABM patients using serum biomarkers is limited [11].

Cerebral microdialysis (MD) is a bedside monitoring technique successfully applied in neuro-intensive care units (NICU) to improve treatment of patients with TBI-, subarachnoid hemorrhage- (SAH) and stroke [12–15]. The technique allows continuous monitoring of the biochemical state of the brain by measuring metabolites in the extracellular fluid (ECF), such as glucose, lactate, pyruvate and glycerol. Glucose, being the main substrate for brain energy metabolism, will through glycolysis become pyruvate, which during normoxic conditions enters the mitochondria and becomes part of the citrate cycle. During hypoxic conditions, energy production will decrease while lactate levels, and the lactate:pyruvate ratio (LPR), will increase [16]. If pyruvate levels remain normal, while lactate levels increase, ongoing mitochondrial dysfunction has been suggested [17].

In the field of TBI, a LPR >40 has been shown to correlate to an unfavorable outcome [18, 19]. Furthermore, a global metabolic crisis, a combination of low glucose and high LPR (Glucose <0.8 mmol/L, LPR >25) in the non-injured hemisphere in TBI patients, has been shown to be an independent predictor of poor outcome [20]. Glycerol, abundant in cell membrane, might be released in the ECF as a sign of ongoing cell death [21] and an arbitrary level of >150 μmol/L has previously been suggested as a pathological threshold [18].

Recently, the metabolic thresholds have been modified suggesting a LPR >30 in combination with pyruvate <70 μmol/L to indicate ischemia, while if pyruvate levels remain high (>70 μmol/L), mitochondrial dysfunction has been suggested [22]. While studies of MD use in ABM patients exist, the potential role of MD, and if deranged metabolism affects cerebral tissue in patients with bacterial meningitis remains unclear [22–26].

As reported previously in the literature, interpretation of biomarkers and MD data may help the clinician to optimize the treatment strategy in cerebral pathologies such as TBI [7], limiting the effect of secondary insults and thus potentially prevent progress of existing brain injury. In line with the above, the aim of this study was to evaluate S100B, NSE and MD parameters in patients with ABM, in order to establish new knowledge into the cerebral energy metabolism and pathophysiology in ABM. To our knowledge, this is the first study to investigate the potential role of biomarkers in combination with MD when monitoring patients with ABM.

## Materials & Methods

The patients included in the present observational cohort study with prospectively gathered data are a subgroup (n = 21) of MD monitored patients from a previous study (n = 52) [4] evaluating ICP targeted therapy in patients with ABM. MD catheter implantation was random and decision was made by the neurosurgeon on call.

### Ethics

The “Regional Ethics Committee Stockholm—Karolinska Institutet” approved the study (Ethical application #2004-1085/1). Informed consent was obtained from relatives to patients included. Inclusion in the study had to be done directly upon admission, with relatives often absent. Consequently, written informed consent could not always be obtained in the intervention group upon admission. If unattainable, a verbal consent by telephone from relatives was obtained and documented in the patient medical records. The local ethics committee approved all procedures. Clinical data is stored in the patient’s hospital charts, which are biometrically protected and stored on hospital servers. The extracted data was analyzed anonymously, with all results presented on a group level, making it impossible to identify individual patients.

### Patient population

Patient inclusion criteria were; (i) age 16–75 years, (ii) confirmed ABM and (iii) admission GCS ≤9, or GCS = 10 combined with lumbar opening pressure >400 mmH_2_O and (iv) intracerebral MD monitoring data available. ABM patients with MD monitoring data >72 hours were included in a subgroup (n = 15) for further analyses. The included patients were treated at the NICU at the Karolinska University Hospital in Stockholm, between January 2008 and December 2010.

### Treatment

The included patients were treated using the basic clinical routine for patients suffering from ABM combined with treatment according to guidelines related to severe TBI, including intracranial pressure monitoring and ICP-targeting therapy in the NICU [4]. The treatment aims at an ICP < 20 mmHg and a cerebral perfusion pressure >50 mmHg. Zero-points for MAP and ICP were the temple. All patients are treated in 30° sitting position. All treatment modalities were registered in the patient monitoring system (Clinisoft^®^; GE Health Care Sverige AB, Danderyd, Sweden). No patient exhibited clinical signs of stroke during the hospitalization. EEG was intermittently performed to exclude seizures.

### Intracranial monitoring

Intracranial pressure (ICP) was monitored with a pressure electrode (n = 2, Codman Microsensor, Depuy Synthes, New Brunswick, NJ, USA) or an external ventricular drain (EVD) (n = 19, Codman, Johnson and Johnson Nordic AB, Stockholm, Sweden). The patients were monitored with a microdialysis device (CMA 70, μDialysis, Stockholm, Sweden) with a cut off membrane of 20 kD, inserted approximately 2 centimetres frontoparietal in the brain parenchyma of the right hemisphere via a separate burr hole. None of the patients was subject to other surgical intervention (i.e. decompressive craniotomy) other than the above for intracranial monitoring.

The catheters were perfused with sterile rinse fluid at a flow rate of 0.3 μl/minute, the estimated recovery rate for glucose and lactate were about 60–70% [27]. Vials with extracted fluid were collected hourly and analysed at a bedside mobile photometric unit (CMA 600, CMA, Sweden) for the following parameters; lactate, pyruvate, glucose and glycerol. From these values, an estimated lactate:pyruvate ratio (LPR) was calculated. All parameters were registered in the ICU-pilot^®^ system (μDialysis AB, Solna, Sweden). The microdialysis value(s) had to be elevated in a minimum of 3 consecutive measurements to be found representative.

Baseline (normal) biochemical levels for the MD parameters were obtained from published data regarding normal human cerebral tissue [28]. The following were used as cut-offs for the analysis; A LPR >40, defined as compromised cerebral metabolism [18], metabolic crisis (LPR >25 and MD-glucose <0.8 mmol/L) [20] and a glycerol level >500 μmol/L. A [20]

Besides ICP and MD monitoring, cerebrospinal fluid (CSF) obtained through the EVD (when applicable) was sampled at least 3 times a week in regards to cell count, glucose, lactate as well as obtaining a CSF culture. The CSF cells and lactate levels were also noted at admission to the hospital. All vital parameters, including laboratory findings, as well as ventilator settings were registered in the ICU-pilot^®^ system (μDialysis AB, Solna, Sweden). Serum levels of glucose were acquired using arterial blood gas samples in the NICU.

### Biomarkers

Samples where obtained from arterial lines in the NICU at admission and every 12 hours (06:00 and 18:00). The peak levels of NSE and S100B shown were all acquired within 72 hours after admission. The samples were analysed using a quantitative automated immunoassay (LIAISON-mat, Diasorin, Italy) for both S100B and NSE.

### Outcome assessment

A board certified physician in neurorehabilitation performed patient follow-up once at 12 to 55 months (median 26 months) after admission. The follow-up included assessment according to Extended Glasgow Outcome Scale (GOSe) [29]. Outcome was dichotomized as favourable (GOSe 7–8) and unfavourable (GOSe 1–6), as previously suggested [20].

### Statistical analyses

The data in tables and text are presented as median +/- interquartile range. Boxplots were used to illustrate, while the Mann-Whitney U Test was used to determine the correlation between MD parameters and biomarkers acquired during the first 72 hours after adission. The biomarker levels had to be acquired during the period the patient was monitored with microdialysis. To correlate MD levels over time the first three days of monitoring, a Wilcoxon matched-pairs signed rank test was used while the data was illustrated as line graphs. Median levels for MD parameters and serum glucose were used 0–24 hours and 48–72 hours after admission. A univariate regression analysis was used to correlate MD and biomarker data with dichotomized outcome (favorable vs. unfavourable) Correlations were calculated using R, version 3.1.0 (The R Foundation for Statistical Computing, Vienna, Austria) and graphs were designed in R and GraphPad Prism, version 6.0 (La Jolla, CA, USA). Differences were considered statistically significant at p<0.05. Raw data used in the analyzes of the 15- and 21 patient cohorts is available (S1 File).

## Results

In the study period, n = 21 patients were included, with a mean age of 47 years (range, 17–74) and n = 11 (52%) of which were males. The patients had an average admission Glasgow Coma Scale (GCS) of 8 (range, 3–10), with eight patients having normal ICP levels (<20 mmHg) while the rest (n = 13, 62%) had a varying degree of increased ICP. CSF cell count was missing in one patient at admission; while two patients had missing CSF lactate levels at admission (missing at random). In 18 patients, S.pneumoniae caused the meningitis, while N. meningitides was the cause in the remaining 3 patients (see Table 1 for demographic data).

**Table 1:** Demographics.

Demographics		n = 21	n = 15
**Male/female**		n = 11/10 (52%/48%)	n = 5/10 (33%/66%)
**Age, years**	median (IQR)	49 (32–61)	50 (40–61)
**Glasgow Coma Scale (GCS) (3–15) at admission**	median (IQR)	9 (7–10)	9 (8–10)
**Pupil unresponsiveness (unilateral)**		n = 4 (19%)	n = 3 (20%)
**CSF white blood cell (WBC, cells per mm**^**3**^**) at admission**	median (IQR)	2,179 (1,293–10,025)	1,900 (1,085–8,700)
	missing	n = 1	n = 0
**CSF lactate at admission (mmol/L)**	median (IQR)	9.0 (8.0–12.0)	9.0 (8.3–11.3)
	missing	n = 2	n = 1
**Increased intra cranial pressure (ICP) during NICU treatment**			
<20 mmHg		n = 8 (38%)	n = 6 (40%)
21–30 mmHg		n = 8 (38%)	n = 7 (47%)
31–40 mmHg		n = 4 (19%)	n = 1 (6.5%)
>40 mmHg		n = 1 (5%)	n = 1 (6.5%)
**Microbiological Agent**			
S. Pneumoniae		n = 18 (86%)	n = 13 (87%)
N. Meningitides		n = 3 (14%)	n = 2 (13%)

Sex, age, admission status, CSF parameters, microbiological agents and the intra cranial pressure are displayed.

### Biomarker correlation to microdialysis parameters

**Fig 1:**
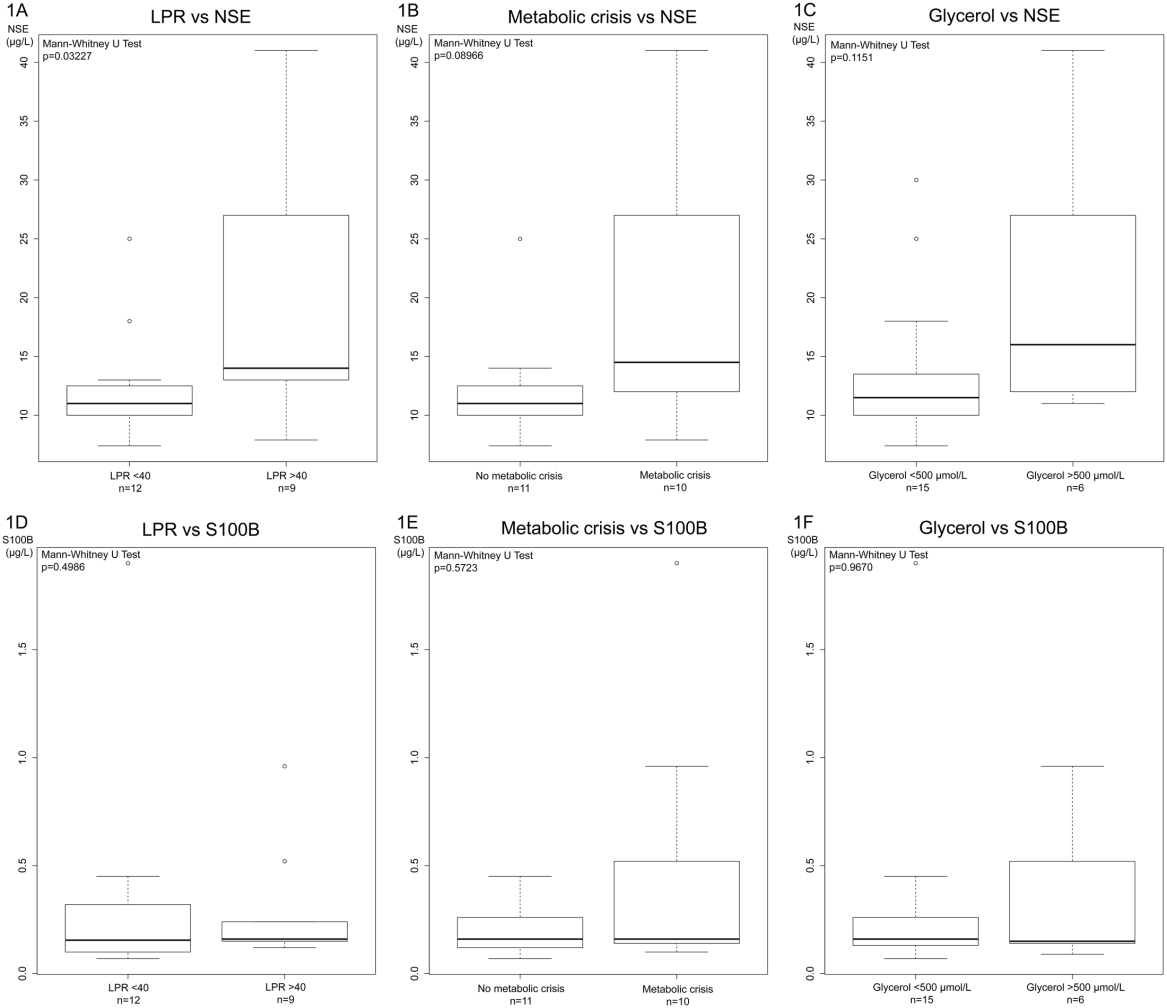
Biomarkers vs MD parameters. Illustrates NSE (A-C) and S100B (D-F) levels in patients (n = 21) with either increased LPR (>40), metabolic crisis (LPR >25, MD-glucose <0.8 mmol/L) and increased glycerol (>500 μmol/L). In general, increased NSE levels were correlated to worse metabolic conditions.

Table 2 summarizes the median peak levels of the biomarkers S100B and NSE in serum (Table 2). NSE was correlated to high LPR levels (>40) (p = 0.03), with similar, although non-significant observations made in patients with high levels of glycerol (>500 μmol/L, p = 0.1) and those with a metabolic crisis (Glucose <0.8 mmol/L, LPR >25, p = 0.9) (Fig 1A–1C). There was no significant correlation between MD parameters and S100B levels (Fig 1D–1F).

**Table 2:** Microdialysis- and biomarker data.

Microdialysis data	n = 21	n = 15 (monitored >72hours)	Reference intervals
***MD parameters*, *including normal cerebral reference intervals***	*median (IQR)*	*median (IQR)*	mean (± SD)
**Glucose (mmol/L),**	1.46 (0.94–2.47)	1.33 (0.88–1.52)	1.7 (± 0.9) mmol/L
**Lactate (mmol/L),**	3.27 (2.61–5.01)	3.01 (2.61–4.34)	2.9 (±0.9) mmol/L
**Pyruvate (μmol/L),**	142 (108–156)	121 (107–148)	166 (±47) μmol/L
**Glycerol (μmol/L),**	142 (60–487)	211 (97–581)	82 (±44) μmol/L
**Lactate:Pyruvate Ratio (LPR),**	25 (22–34)	27 (22–34)	23 (±4)
**LPR >40**	n = 9 (43%)	n = 7 (47%)	
**Glycerol >500 (μmol/L)**	n = 6 (29%)	n = 6 (40%)	
**Metabolic crisis (glucose <0.8 mmol/L, LPR >25)**	n = 10 (48%)	n = 9 (60%)	
	*median (IQR)*	*median (IQR)*	
**S100B, peak serum level (μg/L)**	*0*.*16 (0*.*14–0*.*28)*	*0*.*16 (0*.*15–0*.*32)*	
**NSE, peak serum level (μg/L)**	13 (11–17)	13 (10.5–17)	
**Cerebral ischemia (LPR >30, Pyruvate <70 μmol/L)**	n = 8 (38%)	n = 7 (47%)	
**Non-ischemic metabolic dysfunction (LPR >30, Pyruvate ≥70 μmol/L)**	n = 5 (24%)	n = 3 (20%)	
**No mitochondrial dysfunction (LPR <30)**	n = 8 (38%)	n = 5 (33%)	

Illustrating median microdialysis levels for the first 72 hours for the patients that had MD monitoring for than this time period (n = 15), presented as median +/- intraquartile range (IQR). Nine patients presented with Lactate:Pyruvate Ratio (LPR) >40 while n = 7 had glycerol levels >500 μmol/L. A metabolic crisis, defined as a glucose level <0.8 mmol/L, and a simultaneous LPR >25, existed in n = 9 patients. The reference levels come from unconscious, but otherwise healthy, patients [28].

### Microdialysis samples

Table 2 summarizes the different levels of the brain metabolites glucose, lactate, pyruvate, glycerol and the lactate:pyruvate ratio (LPR) (Table 3). The median LPR was 25 while n = 9 (43%) presented a LPR >40. High glycerol levels (>500 μmol/L) were found in n = 6 (29%). A metabolic crisis, defined as a glucose level < 0.8 mmol/L in combination with a simultaneous LPR > 25, was detected in n = 10 (48%) patients.

**Table 3:** Outcome.

Outcome		n = 21	n = 15
**Time from admission to assessment, days**	Median (IQR)	761 (534–872)	627 (534–856)
**GOSe**	7–8	n = 17 (81%)	n = 12 (80%)
	1–6	n = 4 (19%)	n = 3 (20%)

Outcome for all patients shown as Extended Glasgow Outcome Score (GOSe), divided into “favorable” (GOSe 7–8) and “unfavorable” (GOSe 1–6). Outcome was assessed once, median follow-up time shown.

Furthermore, according to recent definitions proposed by Nordström and colleagues [22], cerebral ischemia (LPR >30, Pyruvate <70 μmol/L) was present in n = 8 (38%), non-ischemic metabolic dysfunction (LPR >30, Pyruvate ≥70 μmol/L) in n = 5 (24%) and no mitochondrial dysfunction (LPR <30) in n = 8 (38%), with similar results reported in their study. In total, n = 13 (62%) suffered from an affected cerebral metabolism according to their definitions.

**Fig 2:**
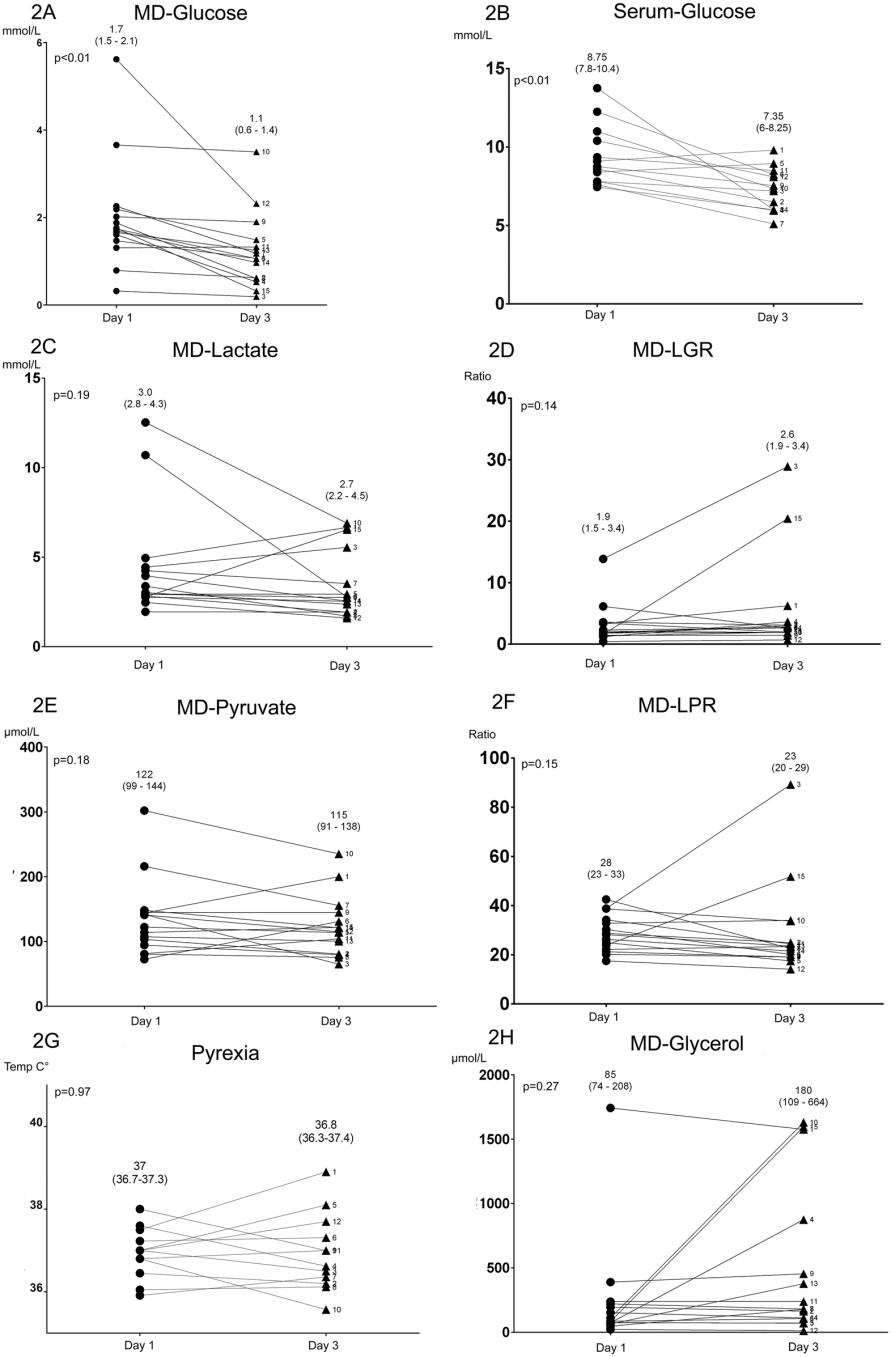
MD parameters, fever and serum glucose over time. Illustrating parameter change over time (median 0–24h, Day 1, vs median 48–72h, Day 3) from insertion of microdialysis (MD) catheters. MD-glucose (2A) and serum-glucose (2B) levels were significantly lower on Day 3 compared to Day 1 (p<0.01 respectively, Wilcoxon matched-pairs signed rank test). No other parameter presented any significant difference over time. For all parameters n = 15 patients are compared except pyrexia (n = 13).

Finally, in a subgroup of n = 15 patients, MD monitoring was available for a minimum of three days, we found that MD-glucose levels (Fig 2A) were significantly higher day 1 compared to day 3 (p = 0.0001). During the same time period, serum glucose level showed a similar significant decrease (p<0.01) (Fig 2B). No other MD metabolite on a group level (Fig 2C–2H) showed any significant difference over time.

### Long-term outcome

Table 3 summarizes the long-term (12–55 months) outcome for all patients (Table 3). In aggregate, a majority of the patients had a favorable clinical outcome, i.e. GOSe 7–8 (n = 17, 81%). There was no statistical significant difference between the groups in regards to MD and biomarker parameters (data not shown). All admission-, biomarker-, ICP-, MD- and outcome parameters are available for each patient (S1 Table).

## Discussion

This observational cohort study analyzes data from 21 patients diagnosed with ABM with biomarker and MD monitoring during their stay and treatment period at the NICU, where several experienced a compromised cerebral metabolism (LPR>40) (n = 9) and/or a metabolic crisis (n = 10). A compromised cerebral metabolism (LPR>40) were correlated to NSE levels (p = 0.03), with similar, although non-significant associations made in patients with high levels of glycerol (>500 μmol/L, p = 0.12) and those with a metabolic crisis (Glucose <0.8 mmol/L, LPR >25, p = 0.09). No significant correlation was found between S100B and MD parameters. Moreover, in patients with more than >72 hours of intracranial monitoring (n = 15), the concentration of glucose in MD samples as well as serum decreased significantly the first days after admission. No significant correlation between MD- or biomarker levels and outcome were found.

In our study, NSE had a better correlation to affected cerebral metabolism measured by MD-parameters than S100B. The correlation between high LPR and serum NSE suggests that extreme anaerobic conditions in the brain affect neurons. However, this does not prove causation as cytokines can both shift to anaerobic metabolism and cause neuronal damage [30]. A previous study by Pleines et al. in TBI patients reported that NSE correlated better to cerebral inflammation than S100B, while S100B was a more accurate marker of cerebral damage [10]. That study revealed that serum levels of the pro-inflammatory cytokine interleukin-6 (IL-6) were correlated to NSE levels, and not S100B. In comparison, the inflammatory component is probably more prominent in ABM than in TBI [31, 32]. Moreover, the permanent cerebral damage in our patient cohort was presumably minimal, since almost all patients had a favorable outcome (81%). However, several minor clinical series and studies have previously demonstrated increased S100B levels at the onset of various CNS infections [11, 33]. While all these studies, including ours, reveal that S100B levels are higher than in healthy controls and mild TBI patients without intracranial pathology [34] (<0.1μg/L), the levels are still comparably relatively low, and it is thus difficult to predict the degree of brain parenchyma damage. In contrast to TBI patients, it is difficult to estimate the exact “beginning” of an ABM. The S100B levels decreased over time, probably since these were taken further from the disease onset compared to S100B levels sampled in TBI patients [7], with a half-life of approx. 30 minutes [35], albeit the kinetics of S100B in serum of meningitis patients is not fully understood. On the other hand, the half-life of NSE is estimated at >20 hours [36] and a plausible explanation why circulating NSE remains in the circulation and is thus correlated to deranged metabolism after admission. Furthermore, the sample size in this, and other studies analyzing biomarker monitoring in TBI, are small resulting in possible underpowered analyses. In aggregate, while S100B and NSE are elevated in serum in ABM as a sign of ongoing injury, NSE seems better correlated to the cerebral biochemical state measured by MD.

The glucose levels significantly decreased over time in both the brain ECF and in serum the first 3 days after admission. Glucose is transported directly from the blood to the ECF through the blood-brain barrier using transporter proteins, and ECF levels are considered more stable than plasma levels due to the transporter proteins ability to adapt to hypo/hyperglycemic conditions [37]. ECF glucose levels have been shown to be around 40% of the serum levels [38], but with high inter-individual variation[39], which is similar to our study. Low levels of glucose are associated with cerebral ischemia, which might be mediated by insufficient supply or possible hyperglycolysis in the injured brain [19, 40]. In ABM, the septic condition might have prompted an increase in catecholamines and cortisol resulting in increased glucose levels [41], and the observations of falling glucose levels in our study might be an expression of a decrease back to the normal physiological conditions, in addition to possible glucose lowering treatment in the NICU. However, as can be seen by Fig 2A some patients had a very low MD-Glucose level, possibly due to bacterial metabolism [42] or tight glucose control at the NICU [43]. A previous study by Schlenk et al. monitored SAH patients with MD and noted that MD-Glucose decreased in patients prior to meningitis diagnosis [26], this being similar to our findings since, as discussed above, it is difficult to estimate the exact beginning of meningitis. In the clinical context, glucose depletion, even without a concurrent increase of lactate, has been associated with an unfavorable outcome [44]. Altogether, glucose decreased in serum and ECF the first days in after admission, perhaps as a normal correction of treatment, albeit infectious- and iatrogenic reasons cannot be ruled out.

The phospholipid glycerol is considered a marker of cell membrane degradation when found in high concentrations in the ECF, and is correlated to an unfavorable outcome [21, 45]. While glycerol levels in our cohort did not significantly increase over time, elevated levels of Glycerol (>150 μmol/L) were found in all but n = 5 patients, with a trend towards correlation with NSE in those of our patients with high levels of glycerol (>500 μmol/L), indicating cellular distress.

Several of our patients had high initial MD-Lactate levels. Previous groups have reported on high levels of lactate in meningitis patients, thought to be caused by increased bacterial anaerobic metabolism in the brain [42]. Usually glucose and lactate is analyzed simultaneously in ABM patients, where increasing lactate and decreasing glucose indicates disease [26]. While we saw lactate levels in the ECF that were higher than in healthy controls, no significant difference of lactate or lactate:glucose ratio over time could be seen in the whole cohort, even if some patients (#3 and #15) clearly indicated these patterns. A rising lactate:pyruvate ratio (LPR >25) with a subsequent decrease of glucose (MD-Glucose <0,8 mmol/L) in seemingly healthy brain tissue after TBI, has been suggested to indicate a metabolic crisis due to anaerobic brain metabolism caused by inadequate blood supply or mitochondrial dysfunction in TBI patients [20, 46]. This pattern was seen in almost 50% of our patients as well. All in all, several of our patients suffered from metabolic crisis and had higher lactate levels than healthy controls, albeit not significantly different over time.

Some of our patients decreased in MD-Pyruvate and increased in LPR (#3 and 15#), although these patterns were not significant on group level. In general, MD-pyruvate levels remained within normal reference levels while LPR was increased (LPR>40) in n = 9 (43%). After an ischemic insult, extracellular pyruvate concentrations may decrease, resulting in an increase of LPR [16]. A reduction of cerebral perfusion in ABM patients has been suggested by Møller et al [47], resulting in a decrease in oxygen and substrate (i.e. pyruvate) delivery, further increasing the LPR reflecting the interstitial shift in redox state. Since n = 13 (62%) of our patients had increased ICP (>20 mmHg) this might also to some extent be suspected in our patient population. Nevertheless, in ABM patients, high levels of pyruvate have also been described by Mazzeo and co-workers [25], believed to represent a specific biochemical effect of the bacterial infection. A recent study by Poulsen et al. proposed different thresholds; ischemia (LPR >30, Pyruvate <70 μmol/L) or non-ischemic metabolic dysfunction (LPR <30, Pyruvate ≥70 μmol/L) [22], both of which were present in our patient cohort in n = 8 (38%) and n = 5 (24%), respectively. Perhaps, in future trials, patients with high LPR could be randomized to strategies that are believed to improve cerebral metabolism, such has hyperbaric oxygen therapy [48, 49].

We were not able to show any significant correlation between biomarker levels, or MD-parameters, and long-term functional outcome. Similar to the other studies analyzing biomarker and microdialysis data [11, 22, 24–26], the sample sizes are underpowered to draw any conclusions on outcome correlation. Furthermore, the lack of clinical outcome correlation could also be explained by the overall “favorable” outcome in our patient cohort, with no cases of mortality and only 4 patients classed as GOSe 1–6 at follow-up (even in that subgroup, the worst GOSe was 5). However, there were patients in our cohort experiencing comprised metabolic conditions with low glucose, increased glycerol, lactate and LPR levels that previous studies have shown correlated to unfavorable outcome (#15, #10 and #3 more so than others in Fig 2). Interestingly, these patients were GOSe 8, 8 and 5 respectively. So while there are patients where biomarker and microdialysis monitoring may guide treatment, this study shows it’s difficult to make any generalized conclusions. In aggregate, more extensive prospective research, preferably in a multi-center fashion, is recommended to determine the potential clinical value of our findings.

In summary, our findings were in the line with the previous literature, with affected cerebral metabolism observed in the majority of our patients. The biomarker NSE, as surrogate marker of outcome, was increased if the LPR was >40, possibly indicating ongoing cerebral tissue injury. Since due to the limited sample size, no correlation to functional clinical outcome could be made. Moreover, MD could help to guide the physician as to the cerebral metabolic status of the patient, where i.e. glucose levels should be closely monitored since we noted significant decreases over time in some patients.

We believe this study demonstrates new understanding of the pathophysiology and cerebral metabolism in ABM, providing a framework for the NICU monitoring of patients with ABM. Specifically, if the cerebral metabolism is affected, neuronal tissue is deteriorating. Moreover, the method of global microdialysis monitoring of cerebrospinal fluid with high temporal resolution in a closed system might optimize treatment of ABM and minimize the risk of iatrogenic infection in NICU patients [7, 50].

### Limitations

Our cohort is small, making larger comparative analyses underpowered. Nevertheless, the data is randomly selected and collected prospectively, representing the largest ABM population with MD and biomarker monitoring in the literature. Moreover, the bacterial etiology in our patient cohort corresponded to that in larger studies [51], with S. Pneumoniae being the causal agent in 18 (86%) of our patients. However, the functional outcome in the cohort is better than in many other ABM cohorts, as is suggested by the new treatment algorithm with ICP guided therapy [4]. The insertion of MD catheters was at random, we don’t believe there to be any selection bias. The MD and biomarker monitoring period is short, but well characterized and continued as long as a patient had an MD catheter. The outcome assessment was performed 12 to 55 months after admission which is a large variation. There is currently no standardized time when outcome is best assessed in ABM patients. However, as is visible in the S1 Table, the time for outcome assessment did not seem to affect patient outcome.

Since MD- and biomarker monitoring is only implemented in the NICU setting, if patients rapidly improve they would be discharged to an ordinary department. Altogether, several patients were only monitored for a short period reducing the already small sample size (n = 21) to n = 15 if trends over time were to be analyzed. However, it is during the initial time the patient is unconscious in the NICU that MD- and biomarker monitoring can potentially be implemented to detect deterioration and guide therapy. Thus, we do not believe this to be a major limitation. Another aspect that might be a shortcoming is the MD technique. MD values are only representative for a few millimeters of brain tissue corresponding to the location of the MD catheter tip, uncertain whether or not this area is representative for the whole brain [52]. If the catheter was close to a border zone of more affected tissue, this could perhaps explain why some patients had very different MD kinetics. It’s difficult to assess this limitation, but since all patients had their catheters inserted in the right frontal lobe, we do not think this to cause any bias. While NSE and S100B are considered brain specific, extracerebral sources have been shown to release these biomarkers [53, 54]. We do however believe that this is a minor issue in ABM patients compared to i.e. multi-trauma patients. NSE has been shown to increase if there is hemolysis in the sample [53], but the Chemical Laboratory at Karolinska University Hospital does not analyze samples if the grade of hemolysis is >0.5 g/L hemoglobin. Altogether, we believe that this group represents a clinical reproducible cohort of ABM patients with minor limitations. Moreover, it is the first to display monitoring of both MD and biomarkers in a NICU setting.

## Conclusion

In this observational cohort study, we were able to show that an affectedcerebral metabolism, monitored using MD-parameters, is frequently present in patients with ABM. Furthermore, patients with compromised cerebral metabolism (LPR>40) had significantly higher levels of NSE, suggesting ongoing deterioration in affected cerebral tissue. However, the potential clinical impact of MD and biomarker monitoring in ABM patients will need to be further elaborated in larger clinical trials.

## Supporting Information

S1 File Raw data. The raw data used in the analyses of the 15- and 21 patient cohorts.
(ZIP)

S1 Table Individual patient data. Admission-, ICP-, biomarker-, MD- and outcome data for each patient. This also includes number of days prior to admission that the patient suffered from CNS symptoms. The increase in ICP is defined as mild (20–30 mmHg), moderate (30–40 mmHg) and severe (>40 mmHg) as according to the Edinburgh University Secondary Insults Grade (EUSIG) [55]. MD definitions according to Poulsen et al are also mentioned [22].
(XLSX)
